# FAT1 inhibits the proliferation of DLBCL cells via increasing the m^6^A modification of *YAP1* mRNA

**DOI:** 10.1038/s41598-024-62793-7

**Published:** 2024-05-23

**Authors:** Tian-long Wang, Xiao-juan Miao, Yan-rong Shuai, Hao-ping Sun, Xiao Wang, Min Yang, Nan Zhang

**Affiliations:** 1 Department of Medical, People’s Liberation Army The General Hospital of Western Theater Command, Chengdu, 610083 China; 2 Department of Hematology, People’s Liberation Army The General Hospital of Western Theater Command, Sichuan Clinical Research Center for Hematological Disease, Branch of National Clinical Research Center for Hematological Disease, Chengdu, 610083 China; 3 Department of Traditional Chinese Medicine, People’s Liberation Army The General Hospital of Western Theater Command, Chengdu, 610083 China

**Keywords:** FAT atypical cadherin 1 (FAT1), Diffuse large B cell lymphoma (DLBCL), Yes1 associated transcriptional regulator (YAP1), Heterogeneous nuclear ribonucleoprotein D (HNRNPD), m^6^A, ALKBH5, Molecular biology, Oncology

## Abstract

Emerging evidence shows that FAT atypical cadherin 1 (FAT1) mutations occur in lymphoma and are associated with poorer overall survival. Considering that diffuse large B cell lymphoma (DLBCL) is the category of lymphoma with the highest incidence rate, this study aims to explore the role of FAT1 in DLBCL. The findings demonstrate that FAT1 inhibits the proliferation of DLBCL cell lines by downregulating the expression of YAP1 rather than by altering its cellular localization. Mechanistic analysis via meRIP-qPCR/luciferase reporter assays showed that FAT1 increases the m^6^A modification of *YAP1* mRNA 3′UTR and the subsequent binding of heterogeneous nuclear ribonucleoprotein D (HNRNPD) to the m^6^A modified *YAP1* mRNA, thus decreasing the stability of *YAP1* mRNA. Furthermore, FAT1 increases *YAP1* mRNA 3′UTR m^6^A modification by decreasing the activity of the TGFβ-Smad2/3 pathway and the subsequent expression of ALKBH5, which is regulated at the transcriptional level by Smad2/3. Collectively, these results reveal that FAT1 inhibits the proliferation of DLBCL cells by increasing the m^6^A modification of the *YAP1* mRNA 3’UTR via the TGFβ-Smad2/3-ALKBH5 pathway. The findings of this study therefore indicate that FAT1 exerts anti-tumor effects in DLBCL and may represent a novel target in the treatment of this form of lymphoma.

## Introduction

Among the various categories of non-Hodgkin's lymphoma, diffuse large B cell lymphoma (DLBCL) has the highest incidence rate, with a year by year increase in prevalence^1,2^. The proliferative activity of DLBCL cells is positively correlated with poor prognosis^3–5^. Therefore, elucidation of the mechanism underlying the regulation of DLBCL cell proliferation is critical for the development of new molecular targeted drugs to treat this form of lymphoma.

FAT atypical cadherin 1 (FAT1), a member of the cadherin superfamily, has attracted research attention in recent years owing to its role in human cancers. The role of FAT1 in different cancers is considered to be context-dependent and tissue-specific. Although mutations and deletion of *FAT1* are associated with tumorigenesis in some malignancies such as skin squamous cell carcinoma, lung cancer, head and neck squamous cell carcinoma, and oral cancer^6–9^, in other cancers, such as breast carcinoma, colorectal cancer, hepatocellular carcinoma, cervical cancer, pancreatic cancer, and gliomas, FAT1 exhibits an oncogenic role^10–15^. However, the specific role of *FAT1* in lymphoma remains unclear. Recently, it has been reported that *FAT1* mutations occur in lymphoma. For example, Zhao et al. reported the occurrence of *FAT1* mutations in 10% of 21 ocular adnexal marginal zone lymphoma cases^16^. Furthermore, Laginestra et al. reported that *FAT1* was mutated in 39% of 21 peripheral T-cell lymphoma, not otherwise specified, cases, and patients with *FAT1* mutations showed inferior overall survival compared with those with wild-type *FAT1*^17^. These two studies suggest that FAT1 plays a vital role in lymphoma. Considering that DLBCL is the category of lymphoma with the highest incidence rate, this study aims to explore the role of FAT1 in DLBCL.

FAT1 has been reported to reduce nuclear localization of Yes1 associated transcriptional regulator (YAP1) by maintaining Hippo pathway activity, thereby inhibiting tumor proliferation^9,18–20^. It has also been reported that YAP1 knockdown can inhibit the proliferation of DLBCL cells. For example, Zhou et al. reported that high expression of YAP1 is significantly correlated with disease progression and poor prognosis, and knockdown of YAP1 expression suppressed cell proliferation and induced cell cycle arrest in DLBCL cells^21^. Further, Wang et al. reported that YAP1 mediates GPNMB-induced pro-proliferative and anti-apoptotic effects in DLBCL^22^. Therefore, we assume that FAT1 may affect the proliferation of DLBCL by regulating YAP1 in this form of lymphoma.

In this study, we demonstrated that FAT1 inhibits DLBCL cell proliferation by downregulating YAP1 expression and explored the mechanism by which FAT1 decreases YAP1 expression.

## Results

### FAT1 inhibits DLBCL cell proliferation by downregulating the protein level of YAP1

**Figure 1:**
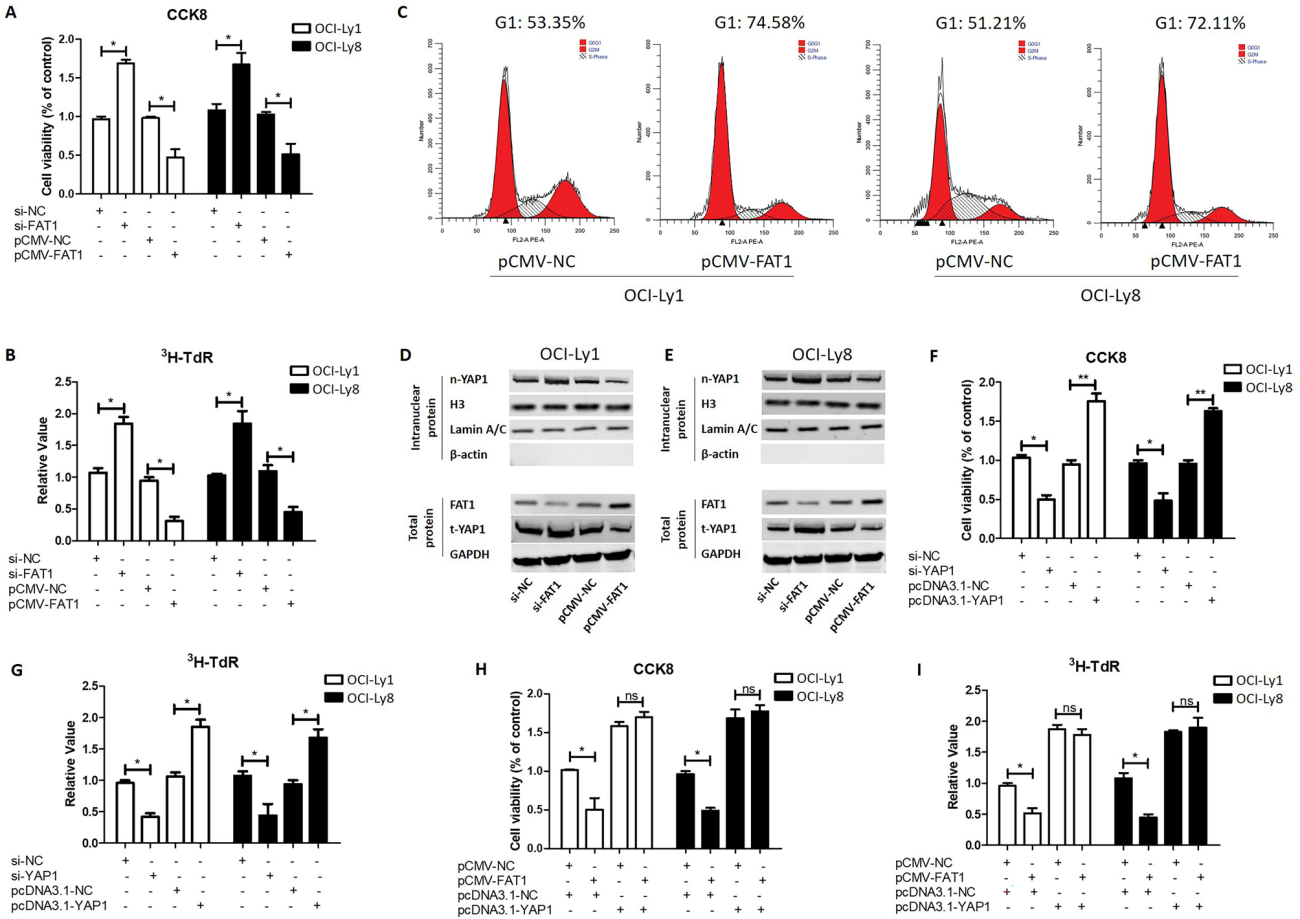
FAT1 inhibits DLBCL cell proliferation by downregulating the protein level of YAP1. (A) OCI-Ly1 and OCI-Ly8 cells were transfected with si-FAT1 (si-NC as control) or pCMV-FAT1 (pCMV-NC as control) for 24 h. Then, cell viability was measured by CCK8 assay. (B) OCI-Ly1 and OCI-Ly8 cells were treated as in (**A**) and then pulsed with 1 mCi of 3H-TdR for 10 h. Subsequently, the cells were collected and assessed for radioactivity. (**C**) OCI-Ly1 and OCI-Ly8 cells were transfected with pCMV-FAT1 (pCMV-NC as control) for 24 h. Then, the cells were collected for flow cytometry. (**D** and **E**) OCI-Ly1 and OCI-Ly8 cells were treated as in (A). Then, the total protein levels of YAP1 and FAT1 were detected by western blot. GAPDH are used as internal reference for total proteins. The intranuclear protein levels of YAP1 were also detected by western blot. H3 and Lamin A/C are used as internal reference for intranuclear proteins. β-actin are used as a cytosolic marker. The infrared imaging and original blots were presented in Supplementary Fig. 1. (**F**) OCI-Ly1 and OCI-Ly8 cells were transfected with si-YAP1 (si-NC as control) or pcDNA3.1-YAP1 (pcDNA3.1-NC as control) for 24 h. Then, cell viability was measured by CCK8 assay. (**G**) OCI-Ly1 and OCI-Ly8 cells were treated as in (**F**); then, cells were pulsed with 1 mCi of 3H-TdR for 10 h. Subsequently, the cells were collected and assessed for radioactivity. (**H**) OCI-Ly1 and OCI-Ly8 cells were transfected with pCMV-FAT1 (or pCMV-NC) and pcDNA3.1-YAP1 (or pcDNA3.1-NC) for 24 h. Then, cell viability was measured by CCK8 assay. (**I**) OCI-Ly1 and OCI-Ly8 cells were treated as in (**H**) and then pulsed with 1 mCi of 3H-TdR for 10 h. Subsequently, the cells were collected and assessed for radioactivity. t: total, n: nucleus, si-FAT1: siRNA for FAT1, si-YAP1: siRNA for YAP1, si-NC: negative control siRNA, pCMV-FAT1: FAT1 expression vector, pcDNA3.1-YAP1: YAP1 expression vector, ns: no significance, *P < 0.05, **P < 0.01.

To investigate the effect of FAT1 on the proliferation of DLBCL cells, we carried out the silencing and over-expression of FAT1 in two DLBCL cell lines, namely, OCI-Ly1 and OCI-Ly8, and performed CCK8 and ^3^H-TdR experiments. The results showed that in the OCI-Ly1 and OCI-Ly8 cells, FAT1 silencing led to increased cell viability, while the overexpression of FAT1 decreased cell viability (Fig. 1A). Similarly, the ^3^H-TdR experiment showed that silencing of FAT1 promoted the proliferation of OCI-Ly1 and OCI-Ly8 cells, while FAT1 overexpression inhibited the proliferation of OCI-Ly1 and OCI-Ly8 cells (Fig. 1B). Flow cytometry results showed that overexpression of FAT1 caused OCI-Ly1 and OCI-Ly8 cells to be arrested at the G1 phase (Fig. 1C). These findings indicate that FAT1 expression exerts an inhibitory effect against DLBCL cell proliferation.

We further explored the mechanism by which FAT1 inhibits the proliferation of DLBCL cells. Consistent with findings reported in other tumors^9,18–20^, we found that FAT1 negatively regulates the intranuclear levels of YAP1 in DLBCL (Fig. 1D,E). Furthermore, our results indicate that FAT1 negatively regulates not only the intranuclear level of YAP1, but also its total protein level in DLBCL (Fig. 1D,E). These findings suggest that, in DLBCL cells, FAT1 regulates the expression of YAP1 rather than its localization as in other tumors. Through CCK8 and H3-TdR experiments, we also confirmed that silencing of YAP inhibited cell proliferation, while overexpression of YAP1 promoted cell proliferation in OCI-Ly1 and OCI-Ly8 cells (Fig. 1F,G). We further explored whether FAT1 inhibits DLBCL cell proliferation by negatively regulating the expression of YAP1. As shown in Fig. 1H,I, when YAP1 was overexpressed, FAT1 no longer inhibited the proliferation of OCI-Ly1 and OCI-Ly8 cells. This result suggests that FAT1 inhibits the proliferation of DLBCL cells by negatively regulating YAP1.

### FAT1 reduces YAP1 protein level via increasing its mRNA m6A level and decreasing its mRNA stability

**Figure 2:**
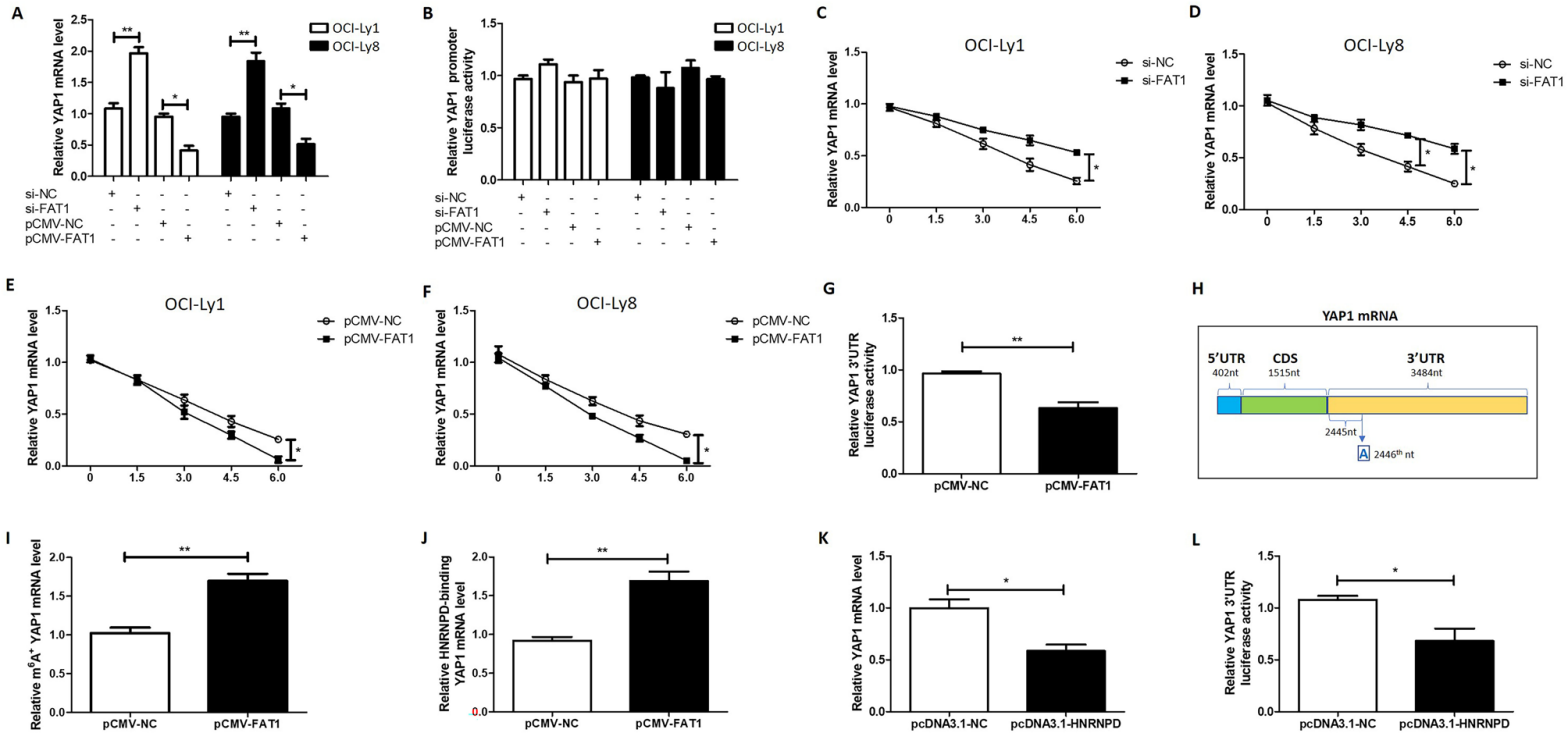
FAT1 reduces YAP1 protein level via increasing its mRNA m^6^A level and decreasing its mRNA stability. (**A**) OCI-Ly1 and OCI-Ly8 cells were transfected with si-FAT1 (si-NC as control) or pCMV-FAT1 (pCMV-NC as control) for 24 h. Then, the mRNA level of *YAP1* was detected by qPCR. (**B**) OCI-Ly1 and OCI-Ly8 cells were grouped and treated as in (A), and the luciferase reporter and pRL-TK were co-transfected. Luciferase activity was measured with the dual-luciferase reporter system. (**C**–**F**) OCI-Ly1 and OCI-Ly8 cells were transfected with si-FAT1 (si-NC as control) (**C** and **D**) or pCMV-FAT1 (pCMV-NC as control) (**E** and **F**) for 24 h, followed by treatment with actinomycin D (Act D, 5 mg/ml) for the indicated duration. Then, the level of *YAP1* mRNA was detected by qPCR. (G) OCI-Ly1 cells were transfected with pCMV-FAT1 (pCMV-NC as control), and the luciferase reporter and pRL-TK were co-transfected. After 24 h, luciferase activity was measured with the dual-luciferase reporter system. (H) The m6A-binding site on the *YAP1* mRNA 3'UTR as predicted by the online website SRAMP. (**I** and **J**) OCI-Ly1 cells were transfected with pCMV-FAT1 (pCMV-NC as control) for 24 h. Then, the cells were collected and the total RNAs were immunoprecipitated using an anti-m^6^A antibody (**I**) or anti-HNRNPD antibody (**J**). Subsequently, the total RNAs were collected and reverse-transcribed, and the mRNA level of *YAP1* was detected by qPCR. (**K**) OCI-Ly1 cells were transfected with pcDNA3.1-HNRNPD (pcDNA3.1-NC as control) for 24 h. Then, the mRNA level of *YAP1* was detected by qPCR. (**L**) OCI-Ly1 cells were grouped and treated as in (**K**), and the luciferase reporter and pRL-TK were co-transfected. Luciferase activity was measured with the dual-luciferase reporter system. si-FAT1: siRNA for FAT1, si-NC: negative control siRNA, pCMV-FAT1: FAT1 expression vector, pcDNA3.1-HNRNPD: HNRNPD expression vector, nt: nucleotide, *P < 0.05, **P < 0.01.

Next, we sought to determine the mechanism by which FAT1 negatively regulates the expression of YAP1. We found that FAT1 silencing upregulated the mRNA level of YAP1, while overexpression of FAT1 downregulated YAP1 mRNA levels (Fig. 2A). These results suggest that FAT1 may affect the expression of YAP1 at the transcriptional or post-transcriptional level. Therefore, we next constructed a luciferase reporter plasmid containing the *YAP1* promoter. The results of the luciferase reporter assay showed that silencing or overexpression of FAT1 did not affect the activity of the reporter plasmid (Fig. 2B), indicating that FAT1 does not regulate YAP1 at the transcription level. Next, we conducted an experiment with actinomycin D to explore whether FAT1 regulates the stability of *YAP1* mRNA. The results showed that when the transcription of *YAP1* was inhibited by actinomycin D, the degradation rate of *YAP1* mRNA in the FAT1-silencing group was slower than that in the control group (Fig. 2C,D). Consistent with this result, the degradation rate of *YAP1* mRNA in the FAT1-overexpression group was higher than in the control group (Fig. 2E,F). These results suggest that FAT1 downregulates YAP1 expression at the post-transcriptional level by decreasing the stability of *YAP1* mRNA.

The 3'UTR of mRNA is critical for mRNA stability; accordingly, we investigated whether FAT1 regulates the stability of *YAP1* mRNA via its 3'UTR. We constructed a luciferase reporter plasmid containing *YAP1* 3'UTR, and performed a luciferase reporter assay. As shown in Fig. 2G, overexpression of FAT1 resulted in a decrease in the activity of the reporter plasmid. This finding indicates that the downregulation of *YAP1* mRNA stability by FAT1 is mediated via the *YAP1* 3'UTR. Extant studies have found that the N6-methyladenosine (m^6^A) modification of 3'UTR exerts a regulatory effect on mRNA stability. Therefore, we next explored whether FAT1 regulates the m^6^A modification of *YAP1* mRNA. We predicted through the online website SRAMP and confirmed that the 3'UTR of *YAP1* mRNA contains an m^6^A binding site at the 2446th nucleotide (nt) (Fig. 2H). Moreover, we used meRIP-qPCR experiments to detect the m^6^A level of the *YAP1* mRNA 3'UTR. As shown in Fig. 2I, the m^6^A level of *YAP1* mRNA 3'UTR was higher in the FAT1-overexpression group than in the control group, indicating that FAT1 upregulates the m^6^A modification of the *YAP1* mRNA 3'UTR.

The above data suggest a causal relationship between the *YAP1* mRNA 3'UTR m^6^A level and 3'UTR-mediated *YAP1* mRNA stability. Existing studies have reported that some RNA-binding proteins bind to m^6^A modified mRNAs and affect their stability. In this study, we found that the RNA-binding protein heterogeneous nuclear ribonucleoprotein D (HNRNPD) binds to the *YAP1* mRNA 3'UTR, as revealed by using the anti-HNRNPD antibody to conduct RIP and subsequently using the primer of amplified *YAP1* mRNA 3'UTR for qPCR and successful amplification (Fig. 2J). Additionally, the binding of HNRNPD to *YAP1* mRNA 3'UTR was further enhanced by overexpression of FAT1 (Fig. 2J). Combined with the finding that FAT1 increased the m^6^A modification of *YAP1* mRNA 3'UTR, the results in Fig. 2J suggest that the FAT1-induced m^6^A modification of *YAP1* mRNA 3'UTR promotes the binding of HNRNPD to this 3'UTR. Furthermore, we found that overexpression of HNRNPD reduced the mRNA level of *YAP1* (Fig. 2K) and the activity of the luciferase reporter plasmid containing the *YAP1* 3'UTR (Fig. 2L). The above results suggest that the increase in *YAP1* mRNA 3'UTR m^6^A levels caused by FAT1 promotes the binding of HNRNPD to the *YAP1* mRNA 3'UTR, thus leading to the decrease in *YAP1* mRNA stability.

### FAT1 increases the m6A level of *YAP1* mRNA by decreasing the expression of *ALKBH5*

**Figure 3:**
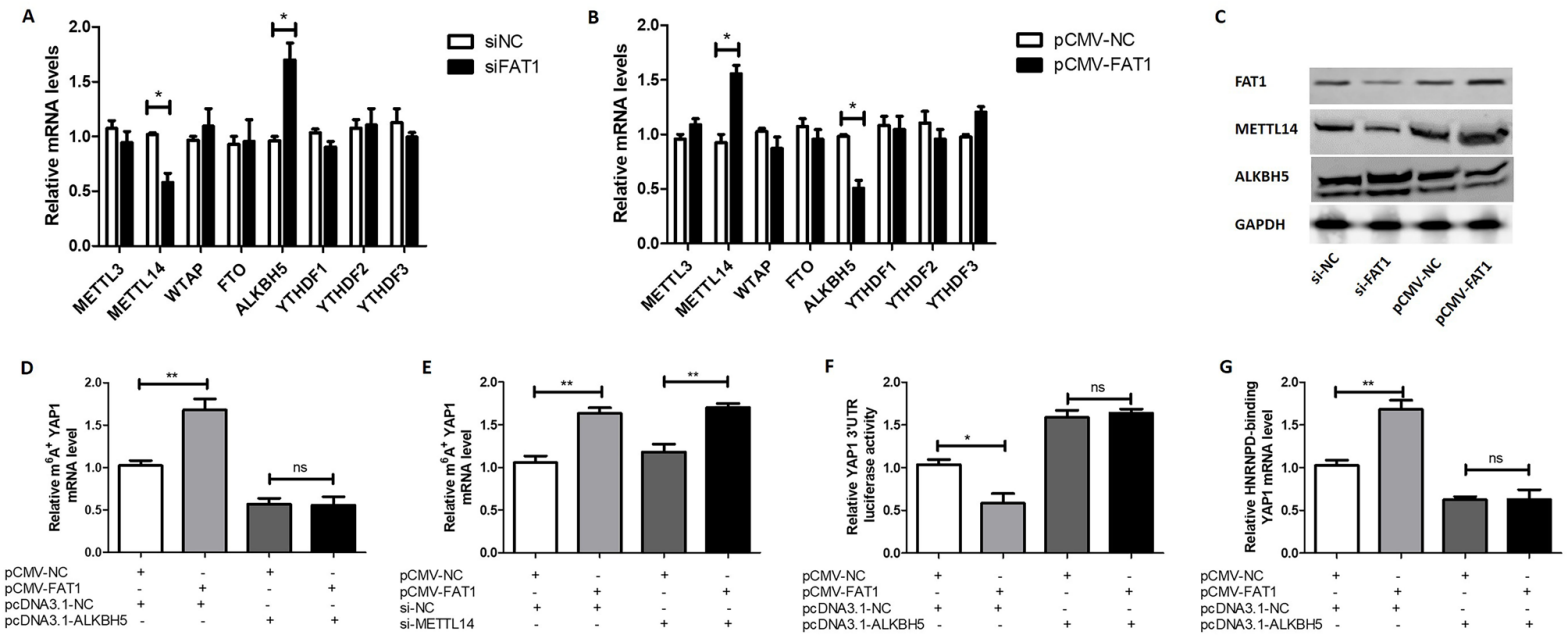
FAT1 increases the m^6^A level of *YAP1* mRNA by decreasing the expression of *ALKBH5*. (**A**–**C**) OCI-Ly1 cells were transfected with si-FAT1 (si-NC as control) (**A** and **C**) or pCMV-FAT1 (pCMV-NC as control) (**B** and **C**) for 24 h. Then, the mRNA levels of eight regulators of RNA m6A modification were detected by qPCR (A, B) and the protein levels of METTL14 and ALKBH5 were assayed by western blot (C). The infrared imaging and original blots were presented in Supplementary Fig. 2. (**D** and **E**) OCI-Ly1 cells were transfected with pCMV-FAT1 (or pCMV-NC) and pcDNA3.1-ALKBH5 (or pcDNA3.1-NC) for 24 h (**D**). OCI-Ly1 cells were transfected with pCMV-FAT1 (or pCMV-NC) and si-METTL14 (or si-NC) for 24 h (**E**). Then, the cells were collected and the total RNAs were immunoprecipitated with an anti-m6A antibody. Subsequently, the total RNAs were collected and reverse-transcribed, and the mRNA level of *YAP1* was detected by qPCR. (F) OCI-Ly1 cells were grouped and treated as in (**D**), and the luciferase reporter and pRL-TK were co-transfected. Luciferase activity was measured with the dual-luciferase reporter system. (**G**) OCI-Ly1 cells were treated as in (**D**). Then, cells were collected and the total RNAs were immunoprecipitated using an anti-HNRNPD antibody. Subsequently, the total RNAs were collected and reverse-transcribed, and the mRNA level of *YAP1* was detected by qPCR. si-FAT1: siRNA for FAT1, si-METTL14: siRNA for METTL14, si-NC: negative control siRNA, pCMV-FAT1: FAT1 expression vector, pcDNA3.1-ALKBH5: ALKBH5 expression vector, ns: no significance, *P < 0.05, **P < 0.01.

The above results show that FAT1 upregulates m^6^A modification of the *YAP1* mRNA 3'UTR. We sought to further explore the mechanism involved. Following overexpression and silencing of FAT1, the mRNA levels of regulators of RNA m^6^A modification, including *METTL3*, *METTL14*, *WTAP*, *FTO*, *ALKBH5*, *YTHDF1*, *YTHDF2*, and *YTHDF3*, were detected. The results showed that silencing of FAT1 downregulated the mRNA level of *METTL14* and upregulated the mRNA level of *ALKBH5* (Fig. 3A), while overexpression of FAT1 upregulated the mRNA level of *METTL14* and downregulated that of *ALKBH5* (Fig. 3B). We further explored the effect of FAT1 on METTL14 and ALKBH5 protein levels, and found the results to be consistent with those for the mRNA levels (Fig. 3C). Therefore, we assume that FAT1 upregulates the m^6^A levels of the *YAP1* mRNA 3'UTR by upregulating *METTL14* or downregulating *ALKBH5*. To test this hypothesis, we next separately blocked the expression of METTL14 and ALKBH5 while simultaneously overexpressing FAT1, and then detected the m6A levels of the *YAP1* mRNA 3'UTR. By performing meRIP-qPCR, we found that when *ALKBH5* was overexpressed, FAT1 no longer upregulated the m^6^A modification level of the *YAP1* mRNA 3'UTR (Fig. 3D). However, we found that when *METTL14* was silenced, FAT1 still upregulated m^6^A modification level of the *YAP1* mRNA 3'UTR (Fig. 3E). These results indicate that FAT1 upregulates the m^6^A modification level of the *YAP1* mRNA 3'UTR by downregulating *ALKBH5* rather than upregulating *METTL14*.

Furthermore, we explored whether ALKBH5 mediated the effect of FAT1 on the stability of *YAP1* mRNA. We found that overexpression of ALKBH5 abrogated the FAT1-induced decrease in the stability of the luciferase-*YAP1* 3'UTR fusion mRNA (the luciferase reporter plasmid containing the *YAP1* 3'UTR), via luciferase reporter assay (Fig. 3F). In addition, we found that overexpression of *ALKBH5* abolished the FAT1-induced increase in the binding of HNRNPD to the *YAP1* mRNA 3'UTR via RIP-qPCR experiments (Fig. 3G). In brief, the above results reveal that FAT1 upregulates the m^6^A modification level of the *YAP1* mRNA 3'UTR by downregulating *ALKBH5* expression, which subsequently promotes the binding of HNRNPD to the *YAP1* mRNA 3'UTR and leads to a reduction in *YAP1* mRNA stability.

### FAT1 attenuates *ALKBH5* transcription by inhibiting the TGFβ/Smad pathway

**Figure 4:**
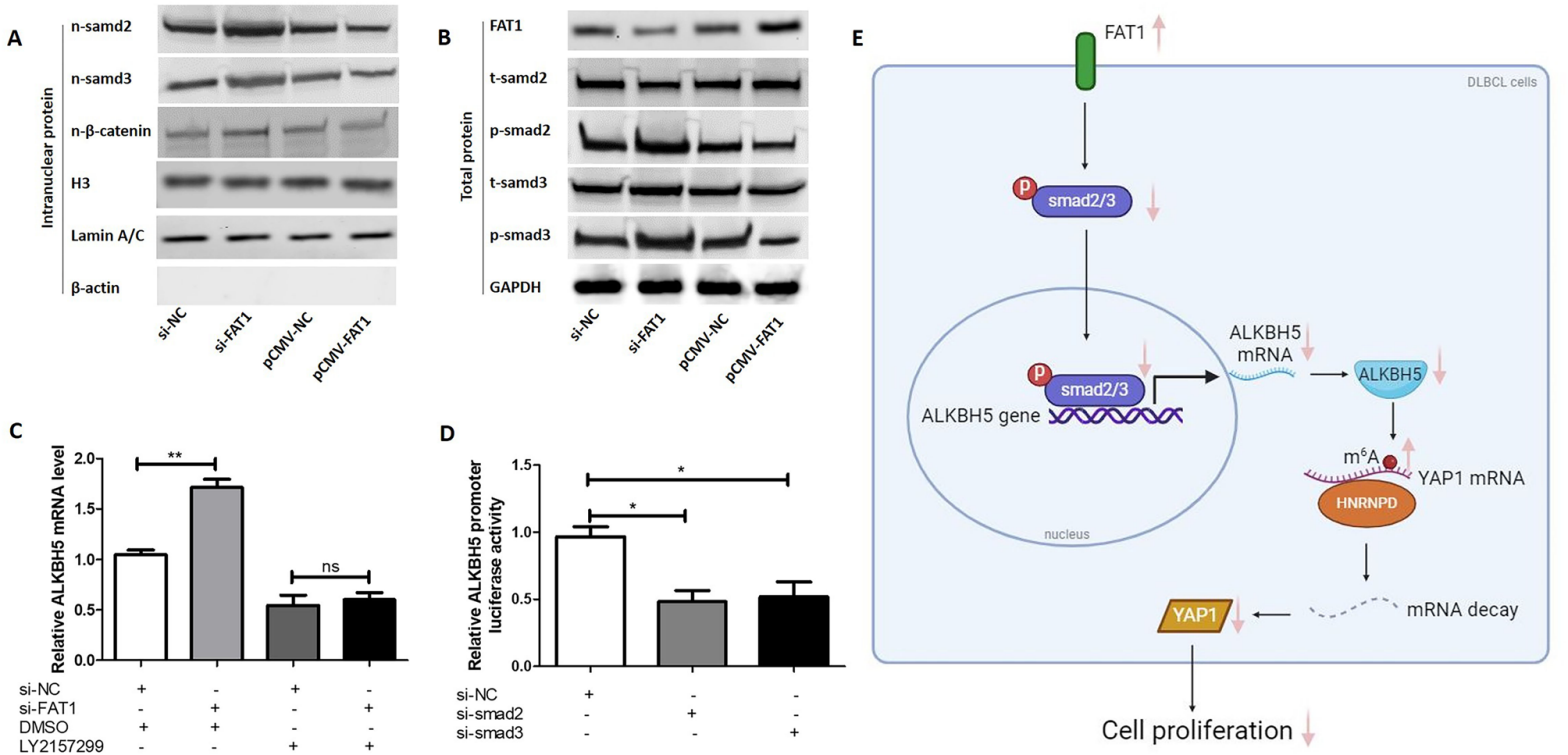
FAT1 attenuates *ALKBH5* transcription by inhibiting the TGFβ/Smad pathway. (**A** and **B**) OCI-Ly1 cells were transfected with pCMV-FAT1 (pCMV-NC as control) or si-FAT1 (si-NC as control) for 24 h. Then, the intranuclear protein levels of smad2, smad3 and β-catenin were detected by western blot. H3 and Lamin A/C are used as internal reference for intranuclear proteins, and β-actin are used as a cytosolic marker (**A**). The total proteins of smad2/p-smad2, smad3/p-smad3 and FAT1 were also detected by western blot. GAPDH are used as internal reference for total proteins (**B**). The infrared imaging and original blots were presented in Supplementary Fig. 3. (**C**) OCI-Ly1 cells were treated with si-FAT1 (or si-NC) and 10 μM LY2157299 (or vehicle control DMSO) for 24 h. Then, the mRNA level of *ALKBH5* was detected by qPCR. (**D**) OCI-Ly1 cells were transfected with si-Smad2 or si-Smad3 (si-NC as control), and the luciferase reporter and pRL-TK were co-transfected. Luciferase activity was measured with the dual-luciferase reporter system. (**E**) graphical summary of explored interactions. t: total, n: nucleus, p: phosphorylated, si-FAT1: siRNA for FAT1, si-smad2: siRNA for smad2, si-smad3: siRNA for smad3, si-NC: negative control siRNA, pCMV-FAT1: FAT1 expression vector, ns: no significance, *P < 0.05, **P < 0.01.

We further explored the possible mechanism underlying the FAT1-induced downregulation of ALKBH5 expression. FAT1 is a membrane receptor that generally plays its role through cell signal transduction. Existing studies have found that FAT1 regulates the WNT and Hippo pathways. The above experiments showed that FAT1 did not affect the localization of YAP1, which reflects the activity of Hippo pathway. Therefore, we focused on the WNT pathway. In addition to the WNT pathway, we focused on the TGFβ/Smad pathway, which also belongs to the developmental biological signal transduction category. The results showed that overexpression and silencing of FAT1 respectively reduced and increased the levels of Smad2/Smad3 proteins in the nucleus, but did not affect the nuclear level of β-catenin (Fig. 4A). We further found that overexpression and silencing of FAT1 also caused a decrease and increase in phosphorylated Smad2/Smad3 proteins, respectively (Fig. 4B). These results indicate that FAT1 may inhibit the activity of the TGFβ/Smad pathway in DLBCL. Next, the TGFβ receptor I inhibitor (LY2157299) was used to inhibit the activity of the TGF/Smad pathway; then, the effect of FAT1 on ALKBH5 expression was examined. The results showed that LY2157299 abolished the increase in *ALKBH5* mRNA levels induced by silencing of FAT1 (Fig. 4C). We further constructed a luciferase reporter plasmid containing the *ALKBH5* promoter region. Luciferase reporter assay results indicated that silencing of Smad2/Smad3 weakened the activity of the reporter plasmid, suggesting that Smad2/Smad3 positively regulate the transcription of *ALKBH5* (Fig. 4D). These results reveal that FAT1 decreases *ALKBH5* transcription by inhibiting the TGFβ/Smad pathway.

## Discussion

FAT1 has been reported to reduce the intranuclear localization of YAP1 by activating the Hippo pathway, thereby reducing the transcription of YAP1 target genes^9,18–20^. Our study confirmed that FAT1 indeed reduces the intranuclear level of YAP1 in in DLBCL. Interestingly, we also found that FAT1 reduced the overall protein and mRNA levels of YAP1, which indicates that the regulation of YAP1 by FAT1 is achieved at the transcriptional level or post-transcriptional level. Further analysis revealed that FAT1 reduced the stability of *YAP1* mRNA, but did not affect its transcription. Therefore, our results show that the regulatory effect of FAT1 on YAP1 in DLBCL occurs at the post-transcriptional level.

FAT1, a cell-membrane receptor, participates in maintaining the activity of the Hippo pathway^18,23^ and inhibiting the WNT pathway^24,25^. However, there are few studies exploring whether FAT1 regulates the TGFβ pathway^26,27^. One study reports that the expression of FAT1 is positively correlated with that of TGFβ1/2 in human gliomas, primary glioma cultures, and other cancer cell lines (U87MG, HepG2, Panc-1, and HeLa cells), and that FAT1 silencing results in decreased expression and secretion of TGFβ1/2^26^. In contrast, another study reports that suppression of FAT1 promotes the nucleocytoplasmic shuttling of TAZ, accompanied by an increase in nuclear Smad3 levels in developing neuroepithelial cells^27^. In this study, we found that FAT1 silencing led to an increase in phosphorylated Smad2/Smad3 and nuclear Smad2/Smad3 levels, while overexpression of FAT1 led to a decrease in phosphorylated Smad2/Smad3 and nuclear Smad2/Smad3 levels; these findings indicate that FAT1 inhibits the activity of the TGFβ pathway in DLBCL.

The TGFβ pathway plays an important role in the regulation of cell growth, differentiation, and development in many biological systems. The regulation of the TGFβ pathway in cancer is complex. This pathway generally plays a tumor-suppressive role in normal tissues and early tumor development. In contrast, in tumor cells, the TGFβ pathway drives tumorigenesis by inducing epithelial-mesenchymal transition, metastasis, angiogenesis, autophagy, and immune suppression^28,29^. The role of the TGFβ pathway in DLBCL is also complex. The newly described TGF-β/Smad1 signaling axis has been reported to be inactivated in more than 85% of DLBCL patients, and DLBCL cell lines lacking Smad1 exhibit a strong growth advantage^30^. Previous studies have reported that the TGFβ pathway plays an oncogenic role^31–33^. For example, Wu et al. reported that DLBCL tissues exhibit high expression of Tim-3, TGF-β, and CXCL12, and this collective upregulation indicates a poor prognosis^31^. Chen et al. reported that CTLA-4 upregulates TGFβ levels in DLBCL, increases the number of stem cells, and enhances proliferation and invasion ability in DLBCL through the TGF-β pathway^32^. Xu et al. reported that TGFβ/Nodal signaling mediates JAM-A-induced increase in cell aggressiveness in DLBCL^33^. In this study, we found that FAT1 inhibited the proliferation of DLBCL by decreasing the activity of the TGFβ-Smad2/3 pathway.

M^6^A modification, one of the most abundant post-transcriptional modifications in mRNA, regulates the metabolic activity of RNA and modulates gene expression^34^. The biological effects of m^6^A modification are dynamically and reversibly regulated by methyltransferases (writers) such as METTL3, METTL14, and WTAP; demethylases (erasers), including FTO and ALKBH5; and m^6^A binding proteins (readers) such as YTHDF1, YTHDF2, and YTHDF3^35^. There are few studies on the role of ALKBH5 and METTL14 in DLBCL^36,37^. Song et al. reported that ALKBH5 decreases m^6^A methylation of the long noncoding RNA *TRERNA1*, thereby upregulating the expression of this mRNA and promoting the cell proliferation of DLBCL, both in vitro and in vivo^36^. Zhang et al. reported that DLBCL patients with high expression of METTL14 exhibit poor survival^37^. In this study, we found that FAT1 downregulated the expression of ALKBH5 and upregulated the expression of METTL14. In addition, ALKBH5 mediated the m^6^A modification of *YAP1* mRNA by FAT1, while METTL14 did not. Whether the FAT1-mediated upregulation of METTL14 plays a role in DLBCL will be investigated in our further studies.

In conclusion, this study reports the novel finding that FAT1 plays an anti-tumor role in DLBCL. Specifically, FAT1 inhibits the proliferation of DLBCL cells by increasing m^6^A modification and decreasing YAP1 expression via the TGFβ-smad2/3-ALKBH5 pathway (Fig. 4E). The results of this study indicate that FAT1 plays an anti-tumor role in DLBCL and may represent a novel target in the clinical treatment of DLBCL.

## Materials and methods

### Cell lines

OCI-Ly1 and OCI-Ly8 cell lines were obtained from the Cell Bank of Type Culture Collection of the Chinese Academy of Sciences (Shanghai, China). Both cell lines were cultured in Modified Eagle Medium (Thermo Fisher Scientific, Waltham, MA, USA) with 10% fetal bovine serum, at 37 °C in a 5% CO_2_ incubator. The genotypic characteristics, phenotypic behavior, and tumorigenic potential of the cells were periodically monitored to confirm identity.

### Quantitative real-time PCR (qPCR)

Total RNA was extracted with Trizol reagent (Comwin Biotechnology, Beijing, China). Then, first-strand cDNA synthesis was carried out using the PrimeScript RT Master Mix kit (TaKaRa, Dalian, China). Next, qPCR was performed using SYBR Select Master Mix (Applied Biosystems, Foster City, CA, USA) according to the manufacturer's instructions, with β-actin as a control. Primer sets for qPCR are listed in Supplementary Table 1.

### Western blot analysis

Total and intranuclear protein was extracted with RIPA (Beyotime, Shanghai, China). Then, protein concentration was measured using the BCA Protein Assay Kit (Beyotime). Next, western blot was performed as described^38^. Finally, The blots were imaged by dual color infrared fluorescence imaging system LI-COR (Odyssey, Lincoln, NE, USA), which requires manual selection of imaging areas during imaging and the imaging process is time-consuming. Therefore, when imaging, we usually chose a limited area membrane instead of the entire membrane for imaging. The infrared imaging and original blots were shown in Supplementary Figs. 1–3. The antibodies anti-FAT1, anti-YAP1, anti-ALKBH5, anti-METTL14, anti-Smad2, anti-Smad3, anti-p-Smad2, anti-p-Smad3, anti-β-catenin, anti-GAPDH, and anti-H3 were obtained from Cell Signaling Technology (CST, Beverly, MA, USA).

### Transfection assay

After growth to 70% to 80% confluence, cells were transfected with siRNAs or expression plasmids using Lipofectamine 2000 (Invitrogen, Carlsbad, CA, USA) for 24 h and used in subsequent experiments. The siRNA sequences are listed in Supplementary Table 2.

### Cell viability assay

Cell viability was determined with the Cell Counting Kit-8 (CCK-8) (Dojindo Laboratories, Kumamoto, Japan), as described^39^. Briefly, DLBCL cells were seeded into 48-well plates for 12 h and subjected to various treatments. Then, the CCK8 reagent was added to the cells (20 ml/well) and the cells were incubated at 37 °C for 1 h. Next, the OD value at 450 nm was determined with a microplate reader (Molecular Devices, Sunnyvale, CA, USA) and normalized against that of the control. The results were presented as cell viability (% of control).

### ^3^H-TdR incorporation assay

DLBCL cells were seeded in triplicate in 96-well plates for 12 h, followed by different treatments. Then, the cells were pulsed in the last 10 h with 1 μCi of 3H-TdR. Next, the cells were collected and assessed for radioactivity using a Tri-Carb 2100TR scintillation analyzer (Perkin Elmer Life Science, Boston, MA, USA). The optical density values of each well represented the proliferation of DLBCL cells.

### Flow cytometry assay

After various treatments, DLBCL cells were trypsinized and fixed in 70% ethanol at 4 °C for 24 h. Then, the cells were incubated with propidium iodide (40 mg/ml) and RNase A (100 mg/ml) (Sigma, St Louis, MO, USA) in phosphate-buffered saline at 37 °C for 1 h. Next, the cells were re-suspended in phosphate-buffered saline for further analysis. Data were acquired using a Beckman Coulter EPICS Elite ESP apparatus (Hialeah, FL, USA) and analyzed using Multicycle AV software (Phoenix Flow Systems, San Diego, CA, USA).

### Plasmid construction

The promoter region or the 3′UTR region were amplified by PCR using genomic DNA from OCI-Ly1 cells as the template. Then, the fragments were separately cloned into a pGL3-Basic vector after digestion with NheI (TaKaRa) and HindIII (TaKaRa). The primer sets for reporter plasmid construction are listed in Supplementary Table 3.

### Luciferase reporter assay

After growth to 70% to 80% confluence in 48-well plates, DLBCL cells were co-transfected with the above luciferase reporters, pRL-TK, and siRNA (or expression plasmid) for 24 h. Next, cell extracts were prepared and luciferase activity was determined using the dual-luciferase reporter system (Promega, Madison, WI, USA) according to the manufacturer's instructions. All experiments were performed three times in triplicate. Firefly luciferase activity was normalized to the *Renilla* luciferase activity. Data were presented as relative luciferase activity over the corresponding control.

### MeRIP/RIP-qPCR

The MeRIP/RIP assay was adapted from a previously reported protocol^40^. Briefly, mRNA was purified from total RNA using PolyATtract mRNA Isolation Systems (Promega Corporation). Next, mRNA was denatured to 70 °C for 10 min, fragmented, and immunoprecipitated with anti-m^6^A antibody or anti-HNRNPD antibody in 1 ml of buffer containing RNasin Plus RNase inhibitor (Promega Corporation), 50 mM Tris–HCl, 750 mM NaCl, and 0.5% Igepal CA-630 (Sigma-Aldrich) for 2 h at 4 °C. Dynabeads® Protein G suspension (Thermo Fisher Scientific) was washed, added to the mixture, and incubated for 2 h at 4 °C with rotation. M^6^A RNA was eluted twice with 6.7 mM N6-methyladenosine 5ʹ-monophosphate sodium salt at 4 °C for 1 h, and precipitated with 5 μg glycogen and one-tenth volume of 3 M sodium acetate in 2.5 volumes of 100% ethanol at − 80 °C overnight. M^6^A enrichment was determined by qPCR. Fragmented mRNA (input) was directly incubated with the above buffer without antibody and treated similarly. The primer set for the fragment containing 2446th nucleotide of the *YAP1* 3′UTR is shown in Supplementary Table 4.

### Statistical analysis

All data are expressed as mean ± SD, unless otherwise stated. Comparisons between two groups were performed with two-tailed unpaired t-tests. Comparisons between three or more groups were carried out with one-way analysis of variance (ANOVA). In all cases, *P* < 0.05 was considered to indicate statistical significance.

### Supplementary Information

Supplementary Figure 1.

Supplementary Figure 2.

Supplementary Figure 3.

Supplementary Tables.

## Data Availability

The datasets generated during and/or analysed during the current study are available from the corresponding author on reasonable request.
