# Supplementary figures and images for: FAT1 inhibits the proliferation of DLBCL cells via increasing the m6A modification of YAP1 mRNA
Source: Sci Rep. 2024 May 23;14:11836. doi: 10.1038/s41598-024-62793-7 (PMC11116375; doi:10.1038/s41598-024-62793-7)

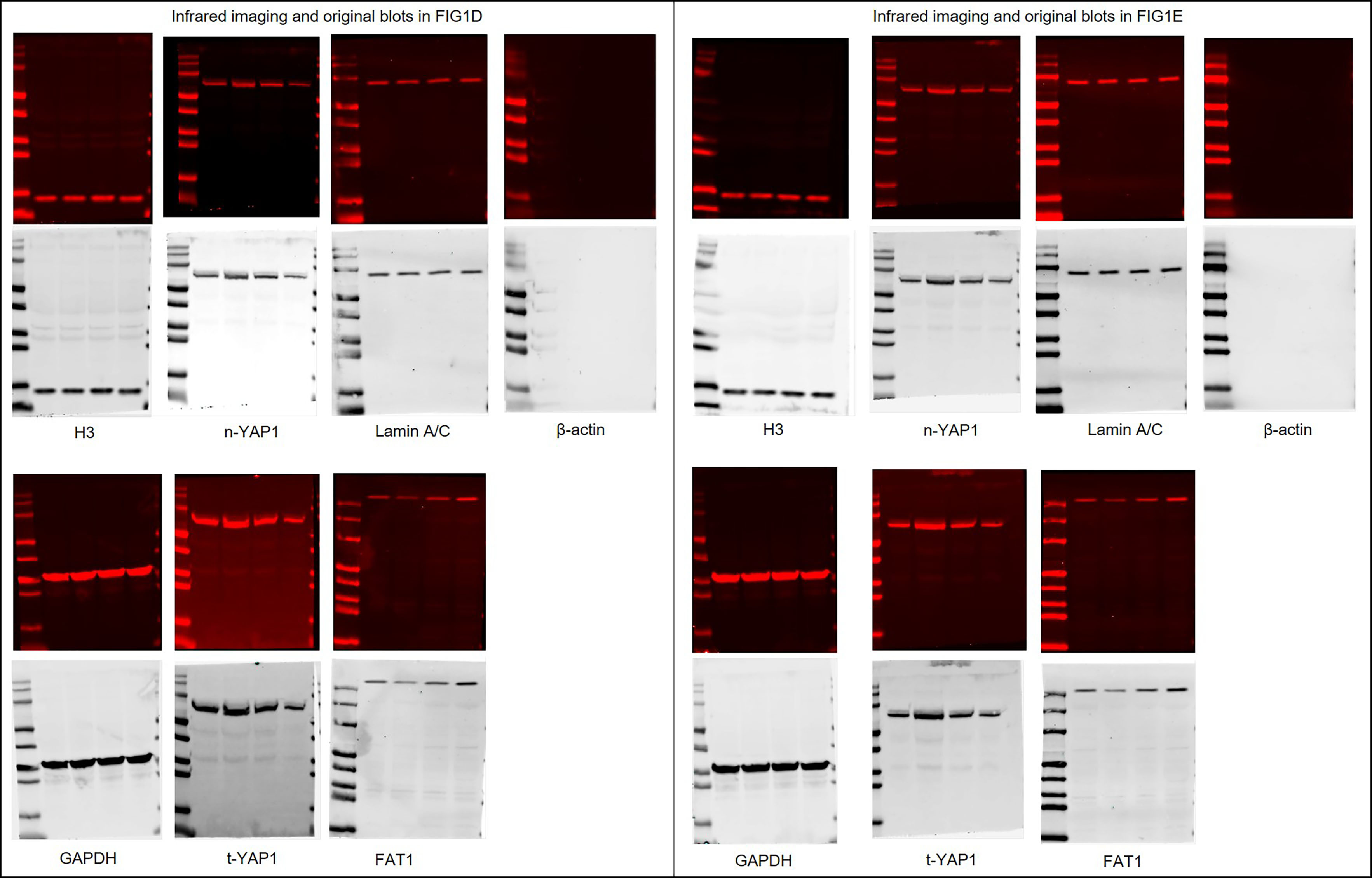

Supplement: Supplementary file 1 — Supplementary Figure 1. [file 41598_2024_62793_MOESM1_ESM.jpg]

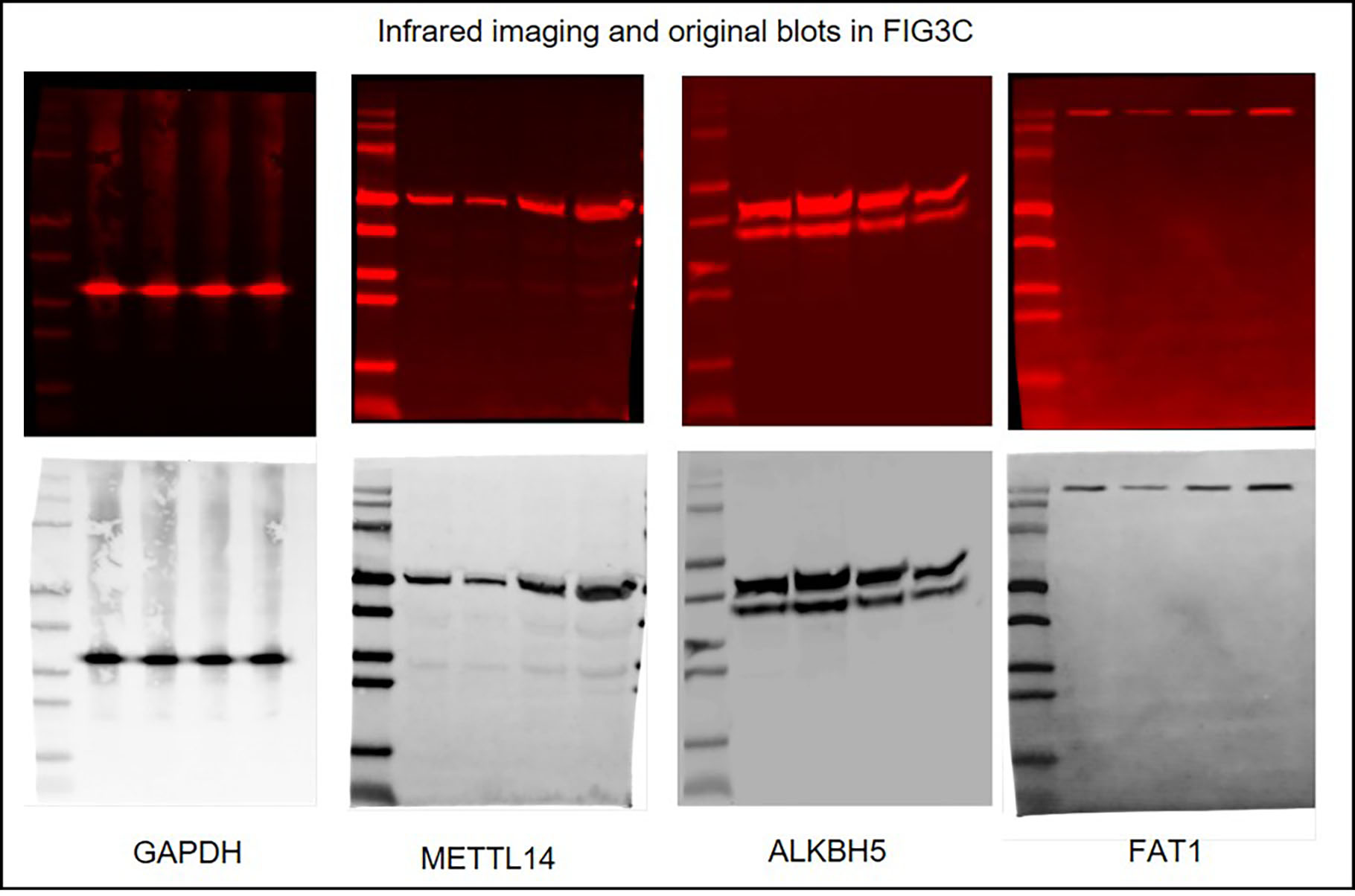

Supplement: Supplementary file 2 — Supplementary Figure 2. [file 41598_2024_62793_MOESM2_ESM.jpg]

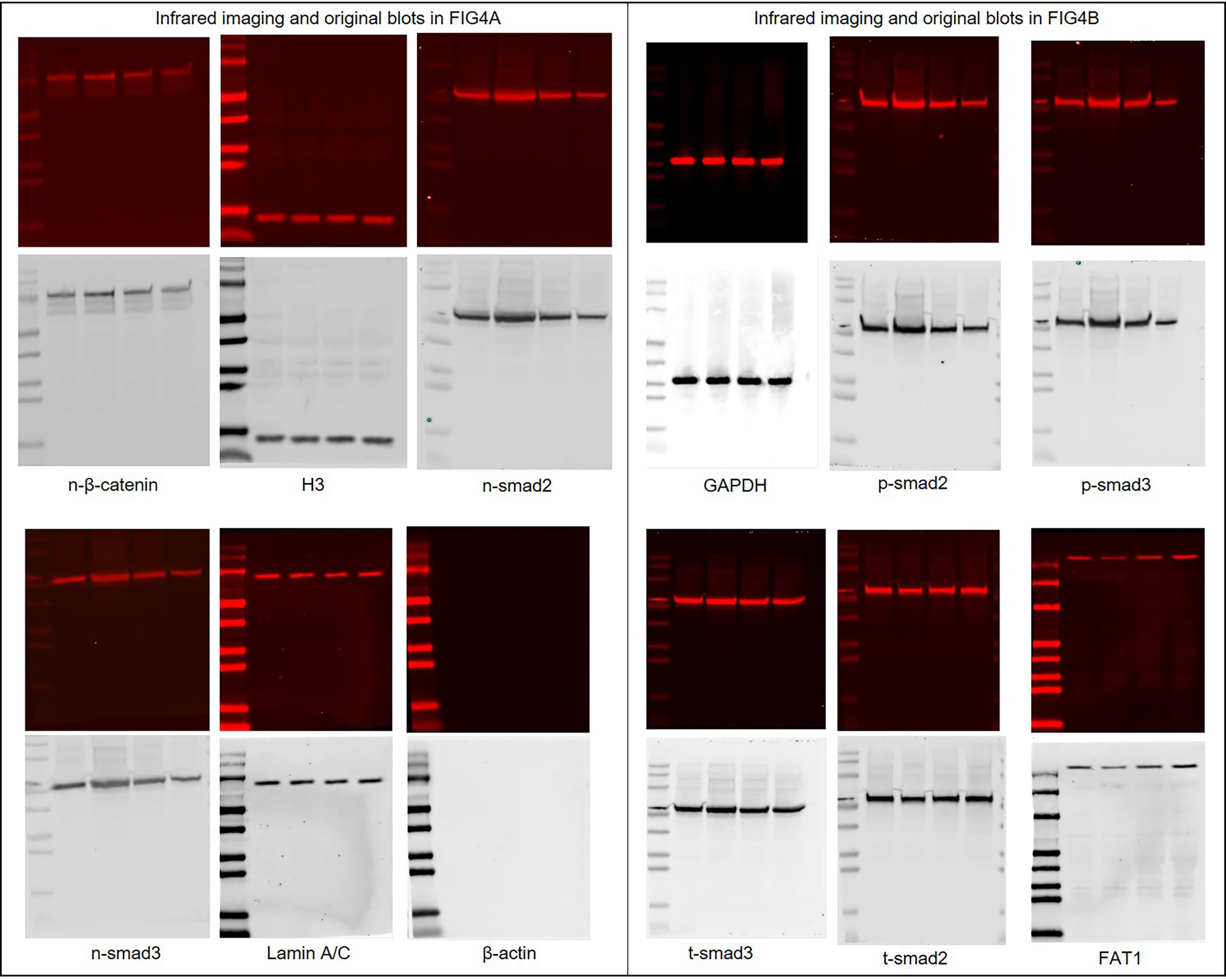

Supplement: Supplementary file 3 — Supplementary Figure 3. [file 41598_2024_62793_MOESM3_ESM.jpg]
