# Supplementary material for: FAT1 inhibits the proliferation of DLBCL cells via increasing the m6A modification of YAP1 mRNA
Source: Sci Rep. 2024 May 23;14:11836. doi: 10.1038/s41598-024-62793-7 (PMC11116375; doi:10.1038/s41598-024-62793-7)
Supplement: Supplementary file 4 — Supplementary Tables. [file 41598_2024_62793_MOESM4_ESM.docx]

**FAT1 inhibits the proliferation of DLBCL cells by increasing** **the m^6^A modification of *YAP1* mRNA**

Tian-long Wang^1,#^, Xiao-juan Miao^2,#^, Yan-rong Shuai^2,#^, Hao-ping Sun^2^, Xiao Wang^2,*^, Min Yang^3,*^, Nan Zhang^2,*^

^1^Department of Medical, People's Liberation Army The General Hospital of Western Theater Command, Chengdu 610083, China

^2^Department of Hematology, People's Liberation Army The General Hospital of Western Theater Command, Chengdu 610083, China

^3^Department of Traditional Chinese Medicine, People's Liberation Army The General Hospital of Western Theater Command, Chengdu 610083, China

**Supplementary Tables**

**Supplementary Table 1.** The primer sets for qPCR

| Gene (human) | Primers |
| --- | --- |
| YAP1 | Forward: 5′-AACTGCTTCGGCAGGCAA-3′ |
|  | Reverse: 5′-GTGTTGGTAACTGGCTACGC-3′ |
| YAP1-3’UTR | Forward: 5′-GAACTCGGCTTCAGGTCCTC-3′ |
|  | Reverse: 5′-GGTTCATGGCAAAACGAGGG-3′ |
| METTL3 | Forward: 5′-TGGTGTCAAAGGAAATCCCCA-3′ |
|  | Reverse: 5′-GACTGGTGGAACGAACCTCA-3′ |
| METTL14 | Forward: 5′-AGTTGGGAGCTGAAAGTGCC-3′ |
|  | Reverse: 5′-TCATAGGAAGCCCTGCAAGT-3′ |
| WTAP | Forward: 5′-CTTCAAAGTTATGGCAAGAGATGAG-3′ |
|  | Reverse: 5′-GGCCAGTTACATCATTAGAGTTAAG-3′ |
| FTO | Forward: 5′-GAGCGCGAAGCTAAGAAACTG-3′ |
|  | Reverse: 5′-GCTGCCACTGCTGATAGAAT-3′ |
| ALKBH5 | Forward: 5′-TCAAGCCTATTCGGGTGTCG-3′ |
|  | Reverse: 5′-AGCAGCATATCCACTGAGCA-3′ |
| YTHDF1 | Forward: 5′-GAGCGCGAAGCTAAGAAACTG-3′ |
|  | Reverse: 5′-GCTGCCACTGCTGATAGAAT-3′ |
| YTHDF2 | Forward: 5′-AGCCTCTTGGAGCAGTACAAA-3′ |
|  | Reverse: 5′-GCATTATTGGGCCTTGCCTG-3′ |
| YTHDF3 | Forward: 5′-ACTAGCGTGGATCAGAGACCT-3′ |
|  | Reverse: 5′-GGAGCCAGGTGATTCTTTGC-3′ |
| β-Actin | Forward: 5′-CGAGGCCCCCCTGAAC-3′ |
|  | Reverse: 5′-GCCAGAGGCGTACAGGGATA-3′ |

| **Supplementary Table 2.** The siRNA sequences | |
| --- | --- |
| Target mRNA | siRNA sequences |
| FAT1 | Forward: 5′-UGGAUUUGUACCAUUCUUCUG-3′ |
|  | Reverse: 5′-GAAGAAUGGUACAAAUCCAAG-3′ |
| METTL14 | Forward: 5′-UGGAUUUGUACCAUUCUUCUG-3′ |
|  | Reverse: 5′-GAAGAAUGGUACAAAUCCAAG-3′ |
| smad2 | Forward: 5′-UGGAUUUGUACCAUUCUUCUG-3′ |
|  | Reverse: 5′-GAAGAAUGGUACAAAUCCAAG-3′ |
| smad3 | Forward: 5′-UGGAUUUGUACCAUUCUUCUG-3′ |
|  | Reverse: 5′-GAAGAAUGGUACAAAUCCAAG-3′ |

**Supplementary Table 3.** The primer sets for PCR amplification of gene promoter or mRNA 3’UTR region

| Amplified region | Primers (the cutting sites of restriction endonucleases are underlined) |
| --- | --- |
| YAP1 promoter | Forward: 5′-CTAGCTAGCTGACCCTCGTTTTGCCATGA-3′ |
|  | Reverse: 5′-CCCAAGCTTCTGCCGAAGCAGTTCTTGC-3′ |
| YAP1 3’UTR | Forward: 5′-CTAGCTAGCGAACTGCTTCGGCAGGCAAT-3′ |
|  | Reverse: 5′-CCCAAGCTTCATCCTGCTCCAGTGTTGGT-3′ |
| ALKBH5 promoter | Forward: 5′-CTAGCTAGCTCACTCACTGCATACGGCCT-3′ |
|  | Reverse: 5′-CCCAAGCTTGGGTGCATCTAATCTTGTCTTCC-3′ |

**Supplementary Table 4.** The primer sets for meRIP-qPCR/RIP-qPCR

| Amplified region | Primer |
| --- | --- |
| Fragment containing 2446^th^ nucleotide of YAP1 3’UTR | Forward: 5′-GAACTCGGCTTCAGGTCCTC-3′ |
|  | Reverse: 5′-GGTTCATGGCAAAACGAGGG-3′ |
